# Three-Dimensional Printed Nanocomposites with Tunable Piezoresistive Response

**DOI:** 10.3390/nano14211761

**Published:** 2024-11-02

**Authors:** Francesca Aliberti, Liberata Guadagno, Raffaele Longo, Marialuigia Raimondo, Roberto Pantani, Andrea Sorrentino, Michelina Catauro, Luigi Vertuccio

**Affiliations:** 1 Department of Industrial Engineering, University of Salerno, Via Giovanni Paolo II, 84084 Fisciano, Italy; lguadagno@unisa.it (L.G.); rlongo@unisa.it (R.L.); mraimondo@unisa.it (M.R.); rpantani@unisa.it (R.P.); 2 Institute for Polymers, Composites, and Biomaterials (IPCB-CNR), Via Previati n. 1/E, 23900 Lecco, Italy; 3 Department of Engineering, University of Campania “Luigi Vanvitelli”, Via Roma 29, 81031 Aversa, Italy; michelina.catauro@unicampania.it

**Keywords:** 3D printed nanocomposites, piezoresistive response, printing direction, tunable gauge factor, two-dimensional sensor

## Abstract

This study explores a novel approach to obtaining 3D printed strain sensors, focusing on how changing the printing conditions can produce a different piezoresistive response. Acrylonitrile butadiene styrene (ABS) filled with different weight concentrations of carbon nanotubes (CNTs) was printed in the form of dog bones via fused filament fabrication (FFF) using two different raster angles (0–90°). Scanning electron microscopy (SEM) and atomic force microscopy (AFM) in TUNA mode (TUNA-AFM) were used to study the morphological features and the electrical properties of the 3D printed samples. Tensile tests revealed that sensitivity, measured by the gauge factor (G.F.), decreased with increasing filler content for both raster angles. Notably, the 90° orientation consistently showed higher sensitivity than the 0° orientation for the same filler concentration. Creep and fatigue tests identified permanent damage through residual electrical resistance values. Additionally, a cross-shaped sensor was designed to measure two-dimensional deformations simultaneously, which is applicable in the robotic field. This sensor can monitor small and large deformations in perpendicular directions by tracking electrical resistance variations in its arms, significantly expanding its measuring range.

## 1. Introduction

Nanocomposites represent a well-established class of materials characterized by superior chemical and physical properties compared to traditional composites [1]. The improved properties imparted by nanofillers have led to the development of a new class of materials suitable for various potential applications [2,3,4,5,6,7]. The addition of suitable nanofillers into the polymeric matrix allows for the creation of multifunctional materials that exhibit additional properties beyond structural support, such as thermal conductivity [8], electrical conductivity [9], and magnetic properties [10]. Among the most used nanofillers, graphene and carbon nanotubes (CNTs) stand out owing to their exceptional electrical properties, making them ideal for creating “smart” materials [11,12,13,14] with multiple functionalities [15,16,17]. CNTs are also very effective in reducing the high sensitivity to chemical and mechanical degradation caused by sunlight exposure [18,19]. One of the significant functionalities is self-monitoring, which has garnered substantial interest in the literature in recent years [20,21]. These sensors are crucial for individual health inspection (e.g., pulse rate, respiratory rate, and swallowing) [22,23], human and robot joint movements [24,25,26], and human–machine interfaces [27]. The manufacturing of conductive nanocomposites can involve processes such as solvent casting [28,29,30], melt mixing followed by compression molding [31,32], and extrusion and injection [33], as well as additive manufacturing [34,35,36,37,38,39]. Additive manufacturing (AM), commonly known as three-dimensional (3D) printing, has been extensively developed in industrial and research fields owing to its clear advantages over subtractive manufacturing methods, particularly for its flexibility and minimal waste generation. The fused filament fabrication (FFF) technique is the most widely used AM method thanks to its low cost, ease of use, reduced number of post-processing steps, and versatility in material usage, including polymers like acrylonitrile butadiene styrene (ABS) [40,41,42], polylactic acid (PLA) [43,44,45], nylon [46,47,48], polycarbonate (PC) [49,50], polyethylene terephthalate (PET) [51,52], and thermoplastic polyurethane (TPU) [53,54].

Optimizing the parameters for achieving functionalities such as self-sensing depends on the manufacturing processes of the composite materials, the micro/nanostructures and conductive materials (size and aspect ratio of the filler), and the geometry of the sample. In a tensile mechanical test, the sample’s increased overall resistance due to load application and subsequent tensile strain is based on the “tunnel effect”. This effect involves electrons being transmitted (or tunneling) between two closely spaced conductive particles through a thin polymer layer, creating a quantum tunneling junction, provided the conductive nanoparticles are within the “tunneling distance” [55]. Additionally, the geometry of the sample, particularly complex geometries achievable through 3D printing, can modulate the sensitivity of the strain sensor by altering the order of the porous structures constituting the printed sample [56]. The effect of printing parameters on the mechanical properties of the 3D printed parts is well documented in the literature. For example, from a mechanical point of view, the printing direction can enhance the strength of the material, especially when the ratio between the nozzle diameter and the printed filament width is less than 1 [57]. Gupta et al. [58] stated that, in the case of 3D printed carbon-based composites, printing direction and filler content are the most important parameters in determining mechanical performance, even more so than printing speed. Hasanov et al. [59] used analysis of variance (ANOVA) to demonstrate that printing temperature and material composition significantly impact the tensile test results of 3D printed samples made of PC/ABS blends. Building orientation and infill density were studied by Gonabadi et al. [60]. The authors found that PLA printed in the on-edge orientation showed a tensile strength of 55 MPa and Young’s modulus of 3.5 GPa, which were about 91% and 40% less for the upright orientation, respectively, demonstrating significant anisotropy. Moreover, the tensile strength and Young’s modulus increased with increasing infill density. The anisotropic effect was also investigated from an electrical point of view. Paz et al. [61] measured the surface electrical conductivity along the X-axis (0°) and Y-axis (90°), and the higher value along the X-axis was attributed to the electrical anisotropy of the samples due to both the preferential orientation of the graphene nanoplates (GNPs) along the printing direction and the directionality of the manufacturing process. Guadagno et al. [15] exploited this effect to develop 3D printed items with regions having different self-heating performances when the whole item underwent the same electrical stimulus (e.g., voltage). Musenich et al. [62] studied the strong dependence of nanocomposite PLA’s mechanical and electrical behaviors on 3D printing pattern orientation through a multi-objective approach. Although anisotropy is documented both from mechanical and electrical points of view, the effects of printing direction and, thus, of anisotropy on the piezoresistive response of nanocomposite materials remain poorly discussed [63].

In this context, the present work aims to investigate the effect of printing conditions, such as raster angle, on the strain-sensing functionalities of CNT-filled ABS under different mechanical testing conditions. A straightforward sensor design is proposed to measure in-plane deformations. This sensor is sensitive to small deformations while accommodating many more significant deformations.

## 2. Materials and Methods

ABS filled with 8% by weight of CNTs, here labeled with the acronym ABS-8%CNTs, was supplied by 3DXTECH Additive Manufacturing in the form of spooled filaments (1.75 mm diameter). ABS was chosen owing to its common use in FFF production, offering good printability, high dimensional stability, and excellent mechanical characteristics [64]. A comparison of ABS with other thermoplastic materials commonly used in the FFF process is reported in Table 1 [65], where for each material characteristic, three levels have been considered: low (L), medium (M), and high (H). From this table, ABS is a good compromise between the ease of printing and the final mechanical properties of the 3D printed object. Even PA and PET show similar characteristics to those of ABS; however, they are semicrystalline polymers that undergo strain-induced crystallization [66,67]. ABS was chosen to investigate only the effect of printing direction on the piezoresistive response of 3D printed materials, excluding any effect of strain-induced crystallization, since it is an amorphous polymer.

To achieve composites with lower filler concentrations, the ABS-8%CNTs masterbatch was diluted with pristine ABS specifically produced for the printing process by SUNLU, California, USA (spooled filament diameter of 1.75 mm). In this case, pristine ABS and pellets of ABS nanocomposite, obtained from the spooled filaments, were blended in a single-screw extruder (3Devo Filament Maker Composer 350, Utrecht, The Netherlands) to obtain the following samples: ABS-0.5%CNTs, ABS-3%CNTs, and ABS-5%CNTs, which were loaded with different weight concentrations of CNTs, i.e., 0.5 wt.%, 3 wt.%, and 5 wt.%, respectively. The temperature profile along the extruder was set to 240/230/230/220 °C for all of the experimental blends. The screw rotation speed was maintained at 5.0 rpm, and the cooling rate was 92%. The resulting filaments had a diameter of 1.75 mm, matching the commercial ones for compatibility with the printing head.

**Figure 1:**
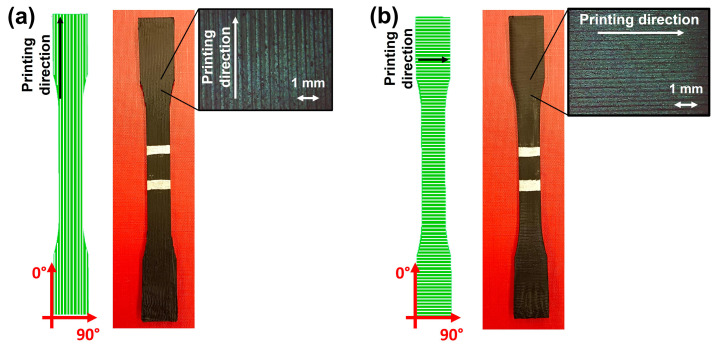
Scheme, image, and optical microscopy of printed samples: (**a**) 0° printing direction; (**b**) 90° printing direction.

The Original Prusa i3 MK2 3D printer (Prusa Research) was used to produce the samples. For mechanical testing, samples were printed with the standard dog bone geometry following the specifications of ASTM D638 standards (American Society for Testing and Materials, ASTM). CAD models were initially designed using Fusion360 software (Fusion360 v.2.0.19941) and then exported as “.stl” files to be loaded into Slic3r Prusa Edition (Prusa Research) software (Slic3r Prusa Edition 2.8.1), which allowed for setting the printing parameters to the values reported in Table S1 of the Supplementary Materials (SM). All samples were printed using identical process parameters, with the exception of the raster angle (printing direction). Specifically, a constant raster angle was maintained throughout the entire thickness of each sample to prevent the formation of a perimeter or external shell, as well as to ensure uniform geometry from the first layer up to the last layer. Two main raster angles were chosen to investigate the effect of this parameter on the self-sensing functionality of the prepared materials: 0° and 90°, as shown in Figure 1. The optical images reported in Figure 1 demonstrate the orientation of deposited filaments in the two different printing directions.

The electrical percolation threshold (EPT) was determined by measuring the electrical conductivity of the samples obtained by nanocomposites with different filler concentrations. Four types of samples were electrically tested: spooled filaments (before printing), single printed filaments (after passing through the thin printing nozzle), and two rectangular-shaped printed samples (30 mm × 10 mm × 3 mm) with raster angles of 0° and 90°, respectively. The electrical conductivity was calculated according to Ohm’s Law, using the electrical resistance obtained from the I-V characteristic. The two-probe method was adopted, as the electrical contacts (see Figure 1) made on the sample surface with silver paint (RS 196-3600, RS PRO, Corby, UK) had negligible resistance compared to the sample resistance, which was on the order of kΩ [16]. A Keithley 6517A multimeter (Solon, OH, USA), configured as a voltage generator, was used to set increasing voltage values, while the electrical current was measured using a 3458A multimeter (Agilent, Santa Clara, CA, USA).

To investigate the morphology of the printed samples, etching treatment was performed to remove part of the polymeric matrix using an oxidizing solution, as detailed in SM. SEM micrographs of the samples were obtained using the SEM LEO 1525 (Carl Zeiss SMT AG, Oberkochen, Germany). More details about sample preparation and the SEM investigation are given in SM. Electrical mapping at the nanoscale level was performed using atomic force microscopy in TUNA mode (TUNA-AFM) to investigate the conductive network composed of CNTs and their spatial configuration. The TUNA current images were analyzed using NanoScope Analysis 1.80 (Build R1.126200; Bruker, Billerica, MA, USA). Table S2 of SM summarizes the optimized parameters for acquiring TUNA current images. Both SEM and TUNA analyses were conducted on the etched samples.

**Figure 2:**
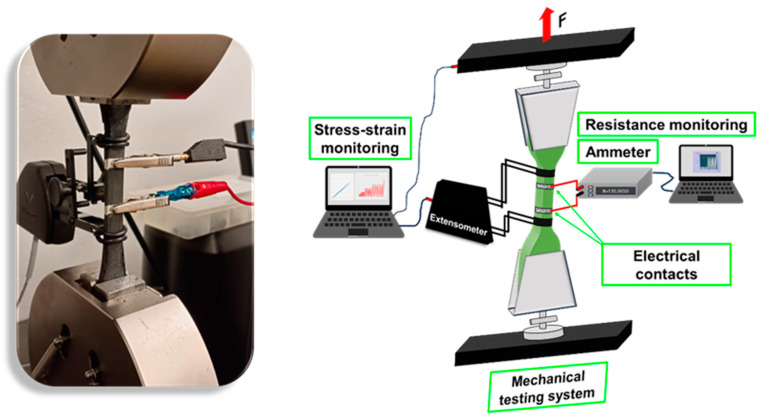
Equipment used to perform mechanical and sensing tests.

The sensing properties were investigated by monitoring the electrical resistance variation during the mechanical tests. The 3458A multimeter (Agilent, Santa Clara, CA, USA) was interfaced with dedicated LabView software (LabView 2020) to record values at a frequency of 2 Hz. Mechanical tests were performed on printed dog bone-shaped samples using a Dual Column Tabletop Testing System (INSTRON, series 5967-INSTRON, Norwood, MA, USA) configured with a crosshead speed of 1 mm/min for tensile and fatigue tests. The fatigue test is a cyclic method specifically designed for the prepared materials, consisting of 5 repetitions (each cycle composed of a loading and unloading phase performed at the same crosshead speed) for each predetermined strain value: 0.25%, 0.5%, 1%, 1.5%, and 2%. To measure the real deformation of the sample during the mechanical test and to exclude any slipping phenomena, local elongation was detected using an axial clip-on extensometer (INSTRON, series 2630-100). Figure 2 shows the equipment used to measure the sample’s resistance variation and mechanical response simultaneously. As shown, the electrical contacts were positioned on the sample within the initial length of the extensometer (central zone of the dog bone-shaped sample) to ensure the detection of resistance variation in the stressed zone. Uniaxial tensile creep tests of the composite films were performed under stress values ranging from 4 to 20 MPa. The applied stress value was determined from the stress–strain plot of the samples to ensure it was within the linear deformation region. The creep and recovery periods were set to 15 min each.

## 3. Results and Discussion

### 3.1. Electrical Properties

**Figure 3:**
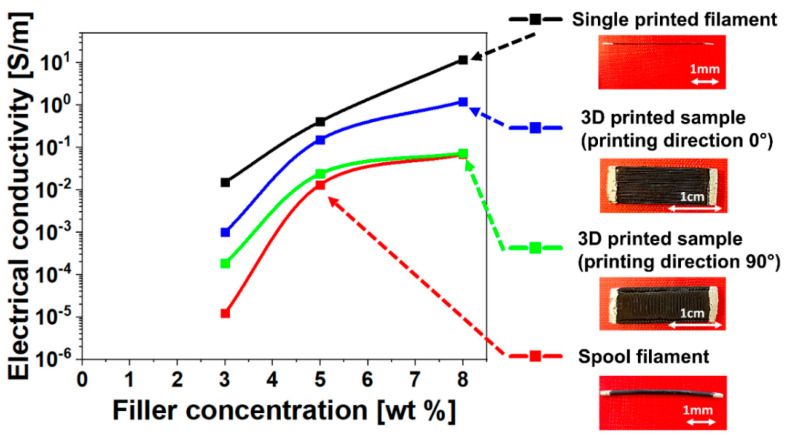
Electrical conductivity of single printed filament, 3D printed samples in both 0° and 90° direction, and the spooled filament for the different investigated CNT concentrations (ABS-3%CNTs, ABS-5%CNTs, and ABS-8%CNTs).

Electrical characterization is the first step in understanding the self-sensing functionality of printed samples. Introducing a conductive filler into an insulating matrix, such as ABS, increases the electrical conductivity, eventually rendering the material electrically conductive. This transition occurs within a range of filler concentrations, where the electrical conductivity exhibits an abrupt increase of several orders of magnitude before reaching a plateau on a logarithmic scale. This behavior, described by a scaling law, is based on the theory of electrical percolation and the tunneling effect [68], where the critical percentage of filler is indicated as the electrical percolation threshold (E.P.T.), i.e., the value beyond which the matrix has a stable electrically conductive network. In our case, it was possible to identify a value of E.P.T. below 5 wt.% for all analyzed systems (see Figure 3).

Figure 3 shows the electrical conductivity versus the amount of filler for the printed samples, single printed filament, and spooled filament. Considering the electrical conductivity of unfilled ABS is on the order of 10^−13^ S/m [69], the introduction of the filler increases the conductivity by several orders of magnitude, reaching a maximum value at 8% concentration, within a range between 10^−2^ and 10 S/m. The observed differences in values depend on the process and printing parameters. Considering the filament alone, the printing process increases the electrical conductivity. However, the organization of the filaments within the printed sample reduces the electrical conductivity. This reduction is more pronounced in samples printed with a raster angle of 90° compared to those printed with a raster angle of 0°. The difference in electrical conductivity values between the spooled filament and the single printed filament is more significant at lower filler concentrations (see Figure 3). The difference ranges from nearly three orders of magnitude at 3 wt.% filler concentration to about two orders of magnitude at 5 wt.% and 8 wt.%. This phenomenon can be explained by the extrusion process, where the spooled filament passing through the nozzle changes the interconnections of the nanofiller along the printed filament, causing a preferential orientation of the carbon nanotubes along the deposition direction. The conductivity curves for the 3D printed samples at different printing directions (0° and 90°) fall within the range of values obtained for the spooled filament and the single printed filament. The conductivity values for the 0° printed samples are closer to those of the single printed filament, while the values for the 90° printed samples are closer to those of the spooled filament. This significant difference between the two configurations is due to the different electrical interconnections of the filaments; a more significant number of electrical interruptions occur in the 90° printed samples compared to the 0° printed samples.

### 3.2. Morphological Characterization

**Figure 4:**
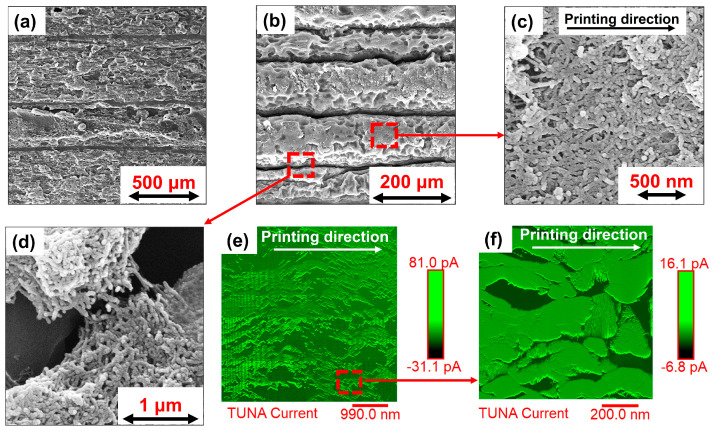
Morphological investigation: (**a**) SEM images of the raw printed sample, (**b**) SEM images of the etched printed sample, (**c**) SEM image of CNTs along a single printed filament, (**d**) SEM images of the inter-filament region, (**e**) TUNA current image along a single printed filament, (**f**) enlargement of TUNA current image.

Figure 4a shows an SEM image of the raw printed sample (8 wt.% CNTs), highlighting the region between the layers of printed filaments. This region becomes more distinguishable when an etching treatment is applied to the sample, as shown in Figure 4b. Figure 4c,d depict SEM images at different magnifications of the single printed filament and inter-filament regions of the produced samples, respectively. Figure 4b, at higher magnification, reveals the separation between adjacent filaments, which is clearly visible owing to the oxidizing treatment that removed the matrix and highlighted the morphology between the filaments. The etching treatment also allowed observation of the arrangement of partially covered carbon nanotubes between different printed filaments, as shown in Figure 4d. The lower compactness of the material between parallel filaments, interspersed by the presence of carbon nanotube contact bridges, explains why the printed sample (printing direction 90°) exhibits reduced electrical conductivity at various filler concentrations when a voltage is applied perpendicular to the printing direction. Conversely, when observing the distribution of carbon nanotubes along the printing direction (as seen in Figure 4c), the filler appears as conductive rails oriented in the printing direction. This configuration clarifies why, despite fewer contacts between adjacent printed filaments, the 0° printed samples show higher electrical conductivity values.

The alignment phenomenon suggested earlier is further supported by the TUNA current images shown in Figure 4e,f, which depict the sample along the printing direction after the etching treatment. Electrically conductive paths, interpenetrating the insulating spatial domains of the matrix, appear on the surface of the observed upper layer with a lighter color tone, indicating regions of higher electrical conductivity. It is evident in Figure 4e,f that CNTs tend to be orientated in the printing direction (white arrow in Figure 4e,f since the lighter colored traces follow the printing direction. Figure S1 (of SM) compares the TUNA current image of the spooled filament (Figure S1a) before the printing process with that of the single printed filament (Figure S1b). Obviously, the spooled filament is conductive thanks to the presence of CNTs and does not show preferential electrically conductive paths. By contrast, a well-oriented electrically conductive path emerges in the TUNA current image of single printed filament due to the CNT alignment along the printing direction (Figure S1b). This distribution increases electrical contacts along the printing direction, thereby contributing to higher electrical conductivity values of the single printed filament (after 3D printing) than the spooled filament (before 3D printing).

### 3.3. Sensing Properties: Tensile Tests

**Figure 5:**
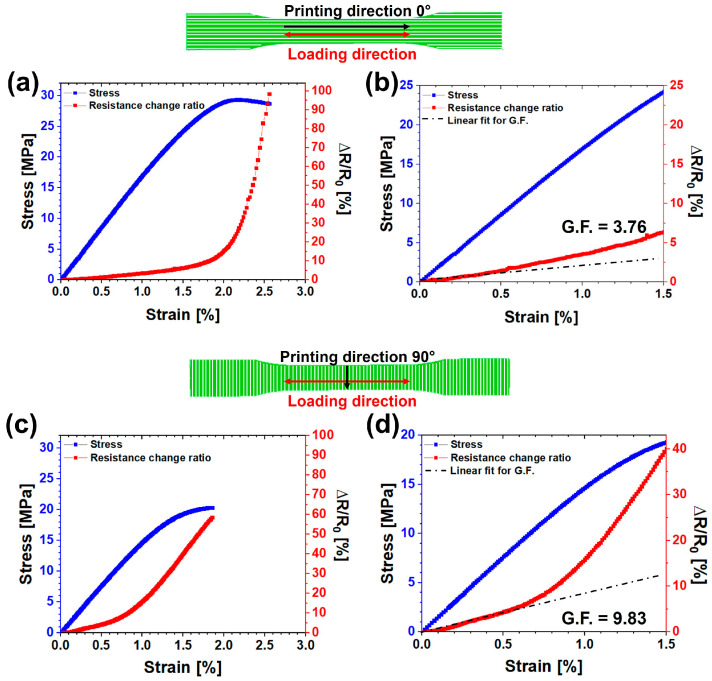
Electrical (red curves and right Y-axis) and mechanical responses (blue curves and left Y-axis) of ABS-5%CNTs samples: (**a**) complete curves of the sample printed in the 0° printing direction, (**b**) enlargement on the elastic regime of the sample printed in the 0° printing direction; (**c**) complete curves of the sample printed in the 90° printing direction, (**d**) enlargement on the elastic regime of the sample printed in the 90° printing direction.

Figure 5 presents the electrical and mechanical responses of the samples containing 5 wt.% CNTs, printed in two directions: 0° (Figure 5a,b) and 90° (Figure 5c,d), measured until failure. The left vertical axis shows the load (stress) plotted against the axial strain, while the right vertical axis depicts the change in electrical resistance expressed as the resistance change ratio ∆R/R_0_ (where ∆R = R − R_0_ represents the variation in resistance relative to the resting-state value R_0_ under no-load conditions).

The sensing properties of strain sensors based on materials filled with carbon filler are attributed to the tunneling phenomenon, which constitutes the primary mechanism for electrical conduction in such structures [68]. The measured resistance of the CNT/polymer nanocomposite arises from both the intrinsic resistance along the CNTs and the inter-tube junction resistance between CNT pairs along the conduction path. The junction resistance consists of resistance due to direct CNT–CNT contact and resistance due to tunneling. By tunneling resistance, we mean the resistance between those CNT pairs that are not at direct contact but are separated by a distance smaller than the electron tunneling gap due to a thin layer of the insulating polymer preventing direct contact between the CNTs. Thus, the electric conduction at CNT–CNT junctions is dominated by tunneling.

The overall electrical resistance depends on parameters such as the number of conducting paths, the distance between conductive particles, the cross-sectional area involved in the tunneling effect, the height of potential barriers between adjacent particles [70], the thickness of the polymer layer between adjacent CNTs, and the polymer properties [71]. Since the tunneling resistance mostly dominates the total electrical resistance, and the change in tunneling resistance is in turn governed by the inter-tube distance variation with applied strain, the tunneling resistance change ratio can be used as a powerful indicator of the strain sensitivity of a CNT/polymer composite. During a tensile test, as the applied load increases elongation, the tunneling distance between adjacent conductive particles also increases, causing breaks in conductive pathways and consequently increasing the bulk electrical resistance.

This behavior typically exhibits a linear trend at low strain values, indicative of the elastic regime, transitioning to exponential behavior at higher strain values where the material enters a fully plastic regime with permanent deformations. This trend is observed in both the printed systems along the 0° and 90° directions (see Figure 5). Focusing on small strain values (see Figure 5b,d), where the system remains in the elastic regime, the sensitivity of the sensor can be quantified by calculating the gauge factor using Equation (1) [6]:

G.F. = (∆R/R_0_)/ε (1)

where ∆R/R_0_ is the resistance change ratio and ε is the measured strain. The gauge factor (G.F.) value is higher for the sample printed in the 90° direction than in the 0° direction, decreasing from 9.83 to 3.76. This difference can be explained by considering the morphological asset of the 3D printed samples. The SEM images reported in Section 3.2 (see Figure 4) evidenced the lower compactness of the materials in the direction perpendicular to the printed filament. It follows that the number of electrical contacts at the interface between adjacent filaments (contact bridges) is lower than the number of electrical contacts along the printed filament, especially if the orientation of the carbon nanotubes is taken into account in this direction [61]. This means that the interface between printed filaments acts as an additional element of inter-tube distancing, thus increasing the tunneling distance (as schematized in Figure S2 of the SM). When a load is applied perpendicular to the printed filaments, the increase in the electrical resistance is mainly due to two reasons: (i) the increase in CNT–CNT distance in each printed filament and (ii) the occurrence of deformations at the interface between filaments where, owing to the lower compactness of the sample printed in the 90° direction, it is more probable that CNT–CNT junctions overcome the electron tunneling gap. On the other hand, when the load is applied parallel to the printed filaments, the interface between the printed filaments plays a less relevant role since the electrical current tends to flow along the oriented carbon nanotubes in the printing direction. In this case, the electrical resistance variation is only due to the increase in the distance of CNT–CNT junctions along the printed filaments. Since the higher the number of CNT–CNT junctions overcoming the tunneling gap, the higher the resistance variation, the ABS-5%CNTs samples printed in 90° are more sensitive to the strain than the ABS-5%CNTs samples printed in 0°, allowing more accurate evaluation of both small and large deformations. This increased sensitivity is also evident at high deformations in the plastic regime, where cumulative irreversible phenomena lead to the destruction of conductive paths. For instance, as seen in Figure 5b,d, a 1.5% elongation results in a 6% resistance variation for the 0° direction, whereas the 90° direction shows a 40% variation.

In addition to the effect of the printing direction on the sensing behavior, even the filler concentration plays a crucial role.

**Figure 6:**
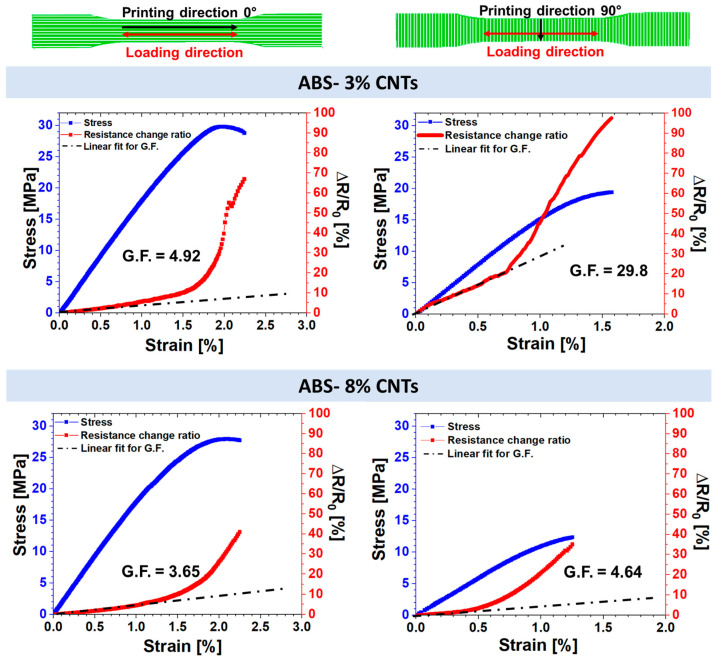
Electrical (red curves and right Y-axis) and mechanical responses (blue curves and left Y-axis) of samples printed in both the 0° and 90° directions for the other two investigated filler concentrations (ABS-3%CNTs and ABS-8%CNTs).

Figure 6 illustrates the electrical and mechanical responses of samples printed in the 0° and 90° directions for the other two investigated filler concentrations (3 wt.% and 8 wt.%).

**Figure 7:**
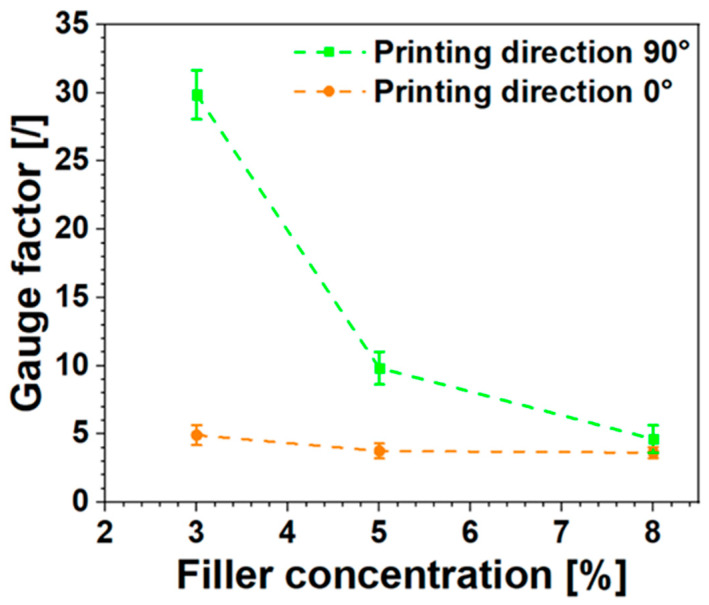
Gauge factor (G.F.) variations with printing direction at different filler concentrations (ABS-3%CNTs, ABS-5%CNTs, and ABS-8%CNTs).

The mechanical response to variations in filler concentration appears to be relatively consistent, with maximum stress and elongation at break values remaining in the range of 25–30 MPa and 2–2.5%, respectively, for samples printed in the 0° direction and 15–20 MPa and 1.5–1.7% for samples printed in the 90° direction, respectively, except for samples filled with 8 wt.%. An excessive concentration of carbon nanotubes of 8 wt.% appears to reduce adhesion between parallel filaments, resulting in the deterioration of mechanical properties. In contrast to the mechanical properties, significant differences are observed in the sensitivity values, expressed by the gauge factor (G.F.), which was evaluated in the linear zone of the ∆R/R_0_ vs. strain curves using Equation (1). These values are summarized and depicted in Figure 7. Changes in the G.F. value can be attributed to variations in the amount of carbon nanotubes.

Figure 7 illustrates the behavior of the G.F. values (calculated similarly as described above) as the filler concentration increases for both types of printed samples.

Sensitivity decreases with increasing carbon nanotube content for both the 0° and 90° direction printed samples. Once again, the dependence of strain sensitivity on the filler concentration is related to the number of CNT–CNT junctions overcoming the tunneling gap when a load is applied to the material. In the case of highly concentrated nanocomposites, the conductive network inside the material is made of a higher number of CNTs than a lower concentrated sample. It follows that when the load is applied, although some CNT–CNT junctions overcome the tunneling gap, leading to an increase in electrical resistance, it is even probable that new CNT–CNT direct contacts could form due to the high density of the conductive network. On the other hand, in lower concentrated nanocomposites, when a load is applied, even if CNT–CNT contacts can form, the probability that this occurs is very low due to the lower filler content. Thus, in this second case, the number of CNT–CNT junctions that overcome the tunneling gap is far higher than the number of forming CNT–CNT contacts. A schematization of the effect of filler concentration on the strain sensitivity is given in Figure S2 of the SM. This explains why the sensitivity of the nanocomposite material to the strain is higher at lower filler content, especially when the filler amount approaches the electrical percolation threshold [72]. In fact, at the same filler content as the 0° printed sample, the less conductive 90° printed sample is nearer to the percolation threshold (see Figure 3) and has higher strain sensitivity.

Furthermore, Figure 7 combines the effect of printing direction with filler concentration. As expected, the difference in sensitivity between samples printed in the 90° direction and those in the 0° direction becomes more pronounced as the carbon nanotube concentration decreases. The variation in gauge factor (G.F.) as a function of carbon nanotube content is less pronounced for samples printed in the 0° direction, decreasing from 4.92 to 3.65. This modest change in G.F. is attributed to the alignment phenomenon of carbon nanotubes, which enhances electrical conductivity in the printed system. By contrast, the variation in filler concentration becomes crucial for samples printed in the 90° direction, as the electrical current flow depends primarily on contact bridges between filaments, which are less effective with lower filler concentrations.

At the same filler content, the different arrangements of CNT–CNT contacts (CNT–CNT contact bridges in the 90° direction versus CNT alignment in the 0° direction) result in different “apparent filler concentrations”. Essentially, in the direction perpendicular to the printing deposition, the system behaves like a material with a lower effective filler concentration than in the longitudinal direction. In light of the discussed results, the printing direction is a new way to vary the sensor’s sensitivity in addition to the traditional methods based on the variation of filler content.

### 3.4. Sensing Properties: Fatigue Test

**Figure 8:**
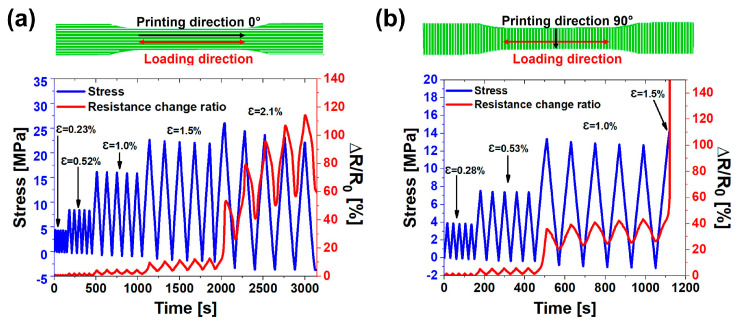
Electrical (red curves and right Y-axis) and mechanical responses (blue curves and left Y-axis) to cyclic tensile loading–unloading tests of ABS-5%CNTs samples printed in (**a**) 0° printing direction and (**b**) 90° printing direction.

In a stress–strain curve, the linear region typically represents the range of elongation values in which the material can regain its original shape without permanent deformation when the system returns to its initial resting conditions. Consequently, the second linear region was not considered in evaluating the gauge factor, as the material exhibits irreversible deformations for strain values higher than 1%. This phenomenon is evident in Figure 8a. The cyclic tensile loading–unloading tests with increasing strain values while monitoring the piezoresistive response of the material were performed on the ABS-5%CNTs samples. Considering the trend of the G.F. values with the filler concentration (see Figure 7), the occurrence of permanent damage just above the elastic regime would be easier to detect in the ABS-3%CNTs samples than in the ABS-8%CNTs samples, even in the case of cyclic tests. As proof of the valuable contribution of fatigue cyclic tests to a better understanding of the elastic limit of the materials, these tests were performed on the ABS-5%CNTs samples only since their electrical conductivity values just above the electrical percolation threshold allowed for obtaining, at the same time, a more sensitive piezoresistive response than the ABS-8%CNTs samples and more reliable and reproducible results than the ABS-3%CNTs samples [73].

Figure 8a shows that for each mechanical cycle, ∆R/R_0_ almost reaches the same maximum value for small deformations, highlighting the repeatability of the resistive behavior of the composites. If the applied strain is less than about 1%, when the material exhibits linear mechanical behavior with the strain, the strength returns to zero after each cycle when the load is removed. Under these conditions, it is possible to state that the system is in a fully elastic regime, meaning that all of the work supplied in the form of elongation is recovered as elastic energy when the system returns to its initial conditions. This indicates that there has been no significant irreversible damage to the structure. Conversely, for strain values greater than 1% (e.g., e = 1.5% and 2.1%), the presence of residual strength indicates the occurrence of permanent damage. Under these conditions, the system can be considered to be in a plastic regime. This phenomenon is due to the rearrangement of the CNT network following plastic deformation in the material. Thus, the micro-damage is related to changes in strength, detectable in a non-destructive manner and not visible to the naked eye [6]. When analyzing the same system obtained with a printing direction of 90° (see Figure 8b), the presence of residual resistance is already detected for strain values equal to 1%. The different behavior is attributed to the reduced “apparent electrical conductivity” due to the lower compactness of the material between one printed filament and another in the 90° printing configuration, as shown in Figure 4. Although in both configurations, the 1% strain falls within the apparent elastic regime (see the stress–strain curves in Figure 5), the presence of intrinsic points of weakness in the printed system with a 90° direction is detected by a residual resistance value different from zero. Specifically, the ABS-5%CNTs system for a strain equal to 1% has no residual resistance if the sample is printed in the 0° direction, while a residual resistance value of approximately 25% is recorded if the printing direction is 90°. In addition, the gauge factor was evaluated along each cycle of fatigue test for both 0° and 90° printed samples. Regarding the ABS-5%CNTs samples printed in the 0° direction, the values of G.F. remain in the range of 3.78–4.06 up to 1% of strain for each cycle. The range of G.F. values during the fatigue test in the elastic regime is perfectly coherent with the G.F. values obtained in the stress–strain tests (see Figure 5b). For the following cycles, just above 1% of deformation since plastic deformations occur in the material, the G.F. does not assume a constant value but tends to increase at each cycle gradually. For the ABS-5%CNTs samples printed in the 90° direction, the G.F. assumes values between 9.05 and 10.47 for all of the cycles at 0.28% and 0.53% of strain. Even in this case, the G.F. values evaluated for these strain cycles in the elastic regime of the material are near the value evaluated in the stress–strain test of 9.83 (see Figure 5d). For the cycles at 1% of deformation, the G.F. values are not constant and tend to increase due to plastic deformations.

In light of these considerations, if only the mechanical properties (stress vs. strain) are analyzed, it is difficult to detect the presence of irreversible damage without an analysis of the electrical behavior following mechanical stress. The electrical signal in response to mechanical stress allows the detection of changes in the structure of the material that conventional stress–strain analysis cannot detect. To better understand this phenomenon, creep and recovery tests were carried out on the same sample (ABS-5%CNTs).

**Figure 9:**
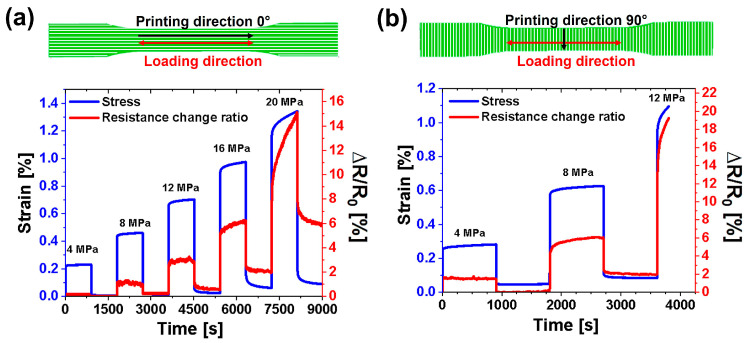
Electrical (red curves and right Y-axis) and mechanical responses (blue curves and left Y-axis) to creep and recovery tests of ABS-5%CNTs samples printed in (**a**) 0° printing direction and (**b**) 90° printing direction.

A creep test is typically used to investigate the mechanical response of materials exposed to a force over a long period. Typically, the creep and recovery curve for a viscoelastic material consists of an initial response corresponding to an elastic instantaneous strain, followed by an elastic-dominant viscoelastic deformation called the primary creep region, and finally, a viscous-dominant viscoelastic deformation called the secondary creep region [74,75]. When the stress is removed, the viscoelastic material recovers from the elastic strain instantaneously and part of the viscoelastic strain slowly. However, the material cannot recover from an amount of strain, called permanent strain, owing to viscous deformation during the creep stage. The creep and recovery tests were carried out at increasing applied load values. As shown in Figure 9a, an increase in applied load causes an increase in permanent strain, which becomes more pronounced by further moving away from the elastic region, identified in this case within a load range up to 10–15 MPa. The ∆R/R_0_ values follow the strain behavior both during the loading phase and the recovery phase, in which a significant residual resistance value is detected when the applied load exceeds 10 MPa. By contrast, the presence of residual resistance is detected even for applied load values lower than 10 MPa when the direction of printing deposition is 90° (see Figure 9b). Similarly to what occurred during the cyclic tests, the presence of weak points in samples printed in the 90° direction can be detected by the presence of a higher value of residual resistance.

### 3.5. Two-Dimensional Sensor Design

So far, the electrical response of printed material has been investigated only in the same direction of applied load. In this section, the printed material piezoresistive response is monitored not only in the same direction as the applied load but also in the perpendicular direction by simultaneously recording two electrical resistance signals. Starting from the previous results, a new sensor design for simultaneously measuring two-dimensional deformations on a plane is presented. The sensor, made of ABS-5%CNTs and printed with a single printing direction, was designed in a cross shape in such a way that the resistance variation along one arm measures the deformation in the parallel direction to the load, while the other arm measures the deformation in the perpendicular direction to the applied load. Thus, when it undergoes deformation, the two-dimensional sensor gives two types of signals with different sensitivities according to the direction of the load applied with respect to the printing direction. Hereafter, three different cases are analyzed: (i) when the load is applied in the same direction of printed filaments, (ii) when the load is applied perpendicularly to the printed filaments, and (iii) when bending stress is applied.

#### 3.5.1. Two-Dimensional Piezoresistive Response When the Load Is Applied in the Same Direction as the Printed Filaments

**Figure 10:**
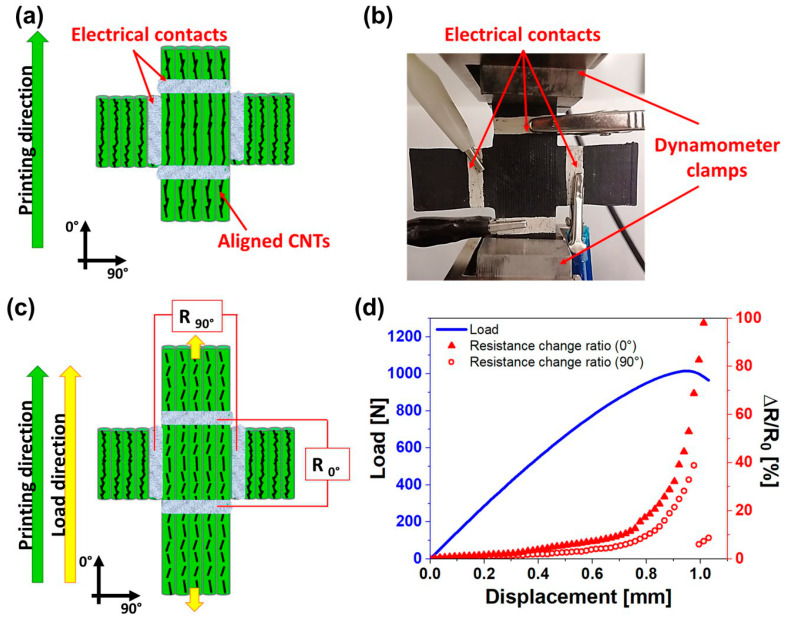
Two-dimensional piezoresistive response when the load is applied in the same direction as the printed filaments: (**a**) scheme of the two-dimensional sensor; (**b**) image of the real sample during the tensile test in the printing direction, (**c**) visualization of material strain and scheme of resistance monitoring during the mechanical test; (**d**) electrical (red curves and right Y-axis) and mechanical responses (blue curve and left Y-axis) of the two-dimensional sensor during the tensile test in the printing direction.

Figure 10a,c reports a scheme of the two-dimensional sensor clarifying the direction of applied stress with respect to the printing direction and the two electrical resistance signals monitored. Printed filaments are aligned in the loading direction, thus ∆R/R_0°_ gives a measure of the deformation in this direction (Figure 10c). At the same time, the deformation occurring in the perpendicular direction to the applied load is detected by ∆R/R_90°_ (Figure 10c). Figure 10b shows the image of the real sample and how it was positioned in the dynamometer while electrical signals were recorded using two ammeters and a dedicated LabVIEW program in a similar way as described in the method section. The two-dimensional piezoresistive response is reported in Figure 10d. According to the results obtained in the previous paragraph, the gauge factor in the 90° printing direction is higher than that in the 0° printing direction. Based on this, it was appropriate to orient the cross so that the 90° printing direction could detect smaller deformations, that is, the strains occurring in the direction perpendicular to the load direction (90° direction in Figure 10c). On the contrary, along the 0° direction in Figure 10c, the printed filaments are oriented in the loading direction. Even in this case, the orientation of CNTs along the printing direction plays a key role. As expected, the electrical resistance along the 0° direction increases due to the tunneling effect (see Figure 10d).

In the 90° direction, from a mechanical point of view, a reduction in the transversal section of the cross-sensor occurs. However, even the resistance change in the 90° direction tends to increase (Figure 10d) due to the fact that electrical signal passes through printed filaments with distanced and aligned CNTs along the load direction, as schematized in Figure 10c. This test demonstrates that it is possible to simultaneously detect both small and high strains occurring in perpendicular directions.

#### 3.5.2. Two-Dimensional Piezoresistive Response When the Load Is Applied Perpendicularly to the Printed Filaments

**Figure 11:**
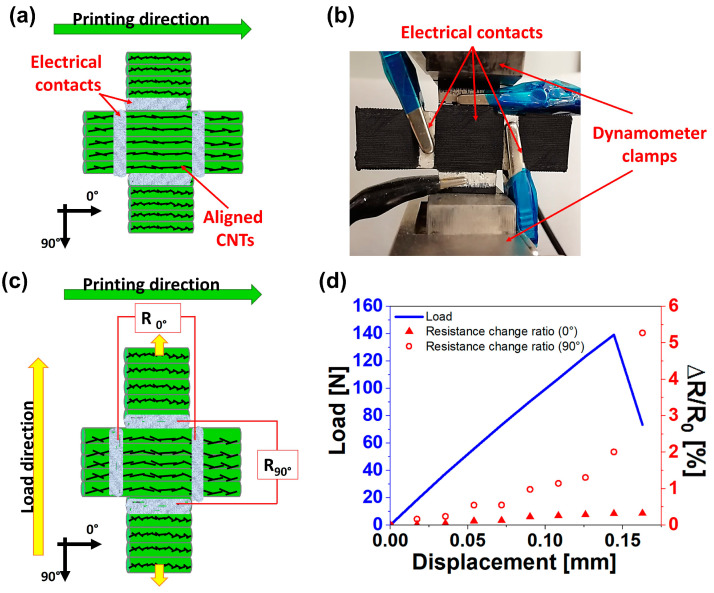
Two-dimensional piezoresistive response when the load is applied perpendicularly to the printed filaments: (**a**) scheme of the two-dimensional sensor; (**b**) image of the real sample during the tensile test in the perpendicular direction to printed filaments, (**c**) visualization of material strain and scheme of resistance monitoring during the mechanical test; (**d**) electrical (red curves and right Y-axis) and mechanical responses (blue curve and left Y-axis) of the two-dimensional sensor the tensile test in the perpendicular direction to printed filaments.

In this case, the cross sensor is positioned in the dynamometer clamps so that the load direction is perpendicular to the printed filaments (see Figure 11a–c. This means that the most sensitive printing direction (90° printing direction) is used to detect the higher strains along the load direction via the ∆R/R_90°_ signal, while the 0° printing direction should detect the smaller strains in the direction perpendicular to the load direction via the ∆R/R_0°_ signal, as schematized in Figure 11c. The two-dimensional piezoresistive response is reported in Figure 11d.

As expected, the small deformations in the direction perpendicular to the applied load are not detected by the 0° printing direction since its piezoresistive sensitivity is low. In fact, no significant changes in the ∆R/R_0°_ signal are recorded during the test (see Figure 11d). Only the deformations along the loading direction are detected by the ∆R/R_90°_ signal, which tends to increase due to the increase in detachment points between adjacent printed filaments. Based on the results of cases (i) and (ii), it is clear that to develop printed sensors, the printing direction has to be set in accordance with the entity of strains to be detected and thus with the loading direction right from the computer-aided manufacturing (CAM) phase of the 3D printing process.

#### 3.5.3. Two-Dimensional Piezoresistive Response When Bending Load Is Applied

**Figure 12:**
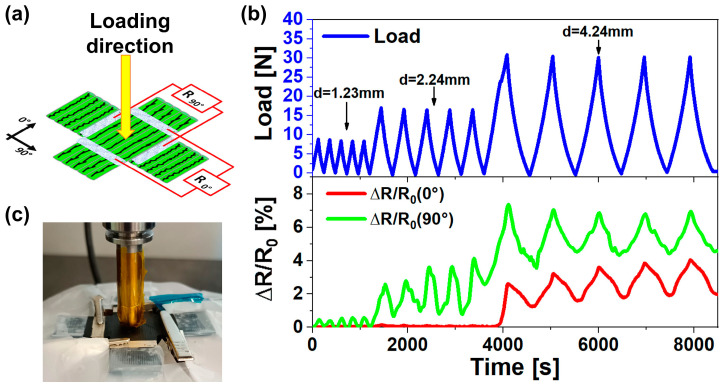
Two-dimensional piezoresistive response when a bending load is applied: (**a**) scheme of resistance monitoring and applied bending load direction on the two-dimensional sensor; (**b**) electrical (red and green curves) and mechanical responses (blue curve) of the two-dimensional sensor the cyclic bending test, (**c**) image of the real sample during the cyclic bending test.

Figure 12a shows a schematization of the cyclic bending test performed on the cross sensor, while Figure 12c shows a photo of the real sample during the test. In this case, the load is applied to the center of the two-dimensional sensor to investigate what happens when deformations of comparable magnitude occur in two perpendicular directions. The two-dimensional piezoresistive response of the cyclic bending test is shown in Figure 12b.

Even in this case, small deformations (in terms of displacement, d) at low loads are detected only along the most sensitive printing direction, which is the 90° printing direction. In fact, at low load values (<15 N), only the ∆R/R90° signal follows the trend of mechanical stress, while the ∆R/R0° signal records no significant changes, although the bending load acts in the same way in both the 0° and 90° directions.

As soon as the applied load reaches the value of 30 N, the ∆R/R_0°_ signal starts to follow the mechanical trend. The higher sensitivity of the 90° printing direction justifies why the ∆R/R_90°_ signal remains higher than the ∆R/R_0°_ signal even at high load values. The relevant aspects arising from this test are, on the one hand, the possibility to broaden the sensitivity range of 3D printed sensors for lower deformations and, on the other hand, to exploit the activation of the ∆R/R_0°_ signal as an alarm threshold, avoiding rupture of the 3D printed part or of the structure in which the 3D printed sensors would be integrated.

Summarizing, the advantages of this two-dimensional strain sensor are threefold. First, the system can easily and simultaneously measure two-dimensional deformation in a plane. Second, the structure of the sensor can be changed without any problem depending on the specific application by properly exploiting the effect of printing direction on the piezoresistive response. Third, the fabrication of this type of strain sensor is simple and provides cost-savings.

### 3.6. Application of Multidirectional Piezoresistive Sensors

**Figure 13:**
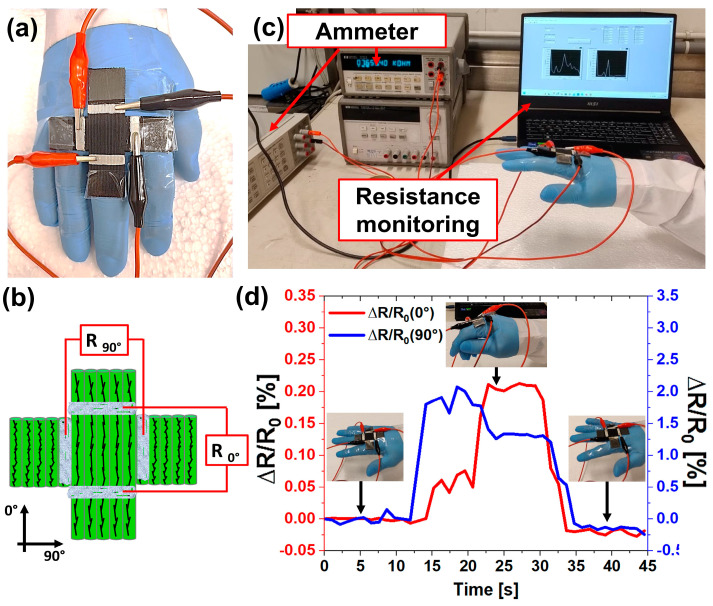
Applications of multidirectional piezoresistive sensors: (**a**) image of the two-dimensional 3D printed sensor applied on the human hand, (**b**) scheme of resistance monitoring and orientation of the two-dimensional sensor, (**c**) scheme of sensing test equipment, (**d**) electrical responses of the two-dimensional sensor applied as a strain sensor of human motion.

This section provides proof of the applicability of 3D printed piezoresistive sensors. The multidirectional strain sensor presented in the previous paragraph was applied to an insulating glove to detect hand movement (Figure 13a). According to the results of the present work, the most sensitive printing direction (90°) was positioned to detect small deformations, as in case (i) of the previous section. In other words, when the hand closes, the main tension occurs along the direction of the finger, while a small deformation occurs at the knuckles. For this reason, the multidirectional sensor was positioned in such a way that the 0° printing direction, having a lower piezoresistive sensitivity, detected the high stress along the finger (∆R/R_0°_) while the small deformations of the knuckles of the hand were felt by the more sensitive 90° printing direction (∆R/R_90°_), as shown in Figure 13a,b. Piezoresistive responses in both directions were recorded in real-time using two ammeters and a dedicated LabView program to record them, as shown in Figure 13c. The results of this representative test of two-dimensional strain sensor application are reported in Figure 13d.

At the beginning of the test, no resistance variations are detected when the hand is utterly open since no movements occur. As soon as the hand starts to close, the resistance variations in the 90° direction show an immediate increase of 0.20%. Although the main movement occurs along the finger, thus along the 0° printing direction, ∆R/R_0°_ starts a few seconds later, reaching lower values (about 0.06%) due to the lower sensitivity of the 0° printing direction than the 90° printing direction. However, when the strain along the finger becomes much higher, ∆R/R_0°_ assumes higher values than ∆R/R_90°,_ proving that the main deformation is acting along the printing direction and thus the hand is quite completely closed (from 22 to 30 s). Finally, when the tensions are released by restoring the initial position of the hand, the piezoresistive response of both directions returns to the initial value. In a more advanced configuration, the two-dimensional piezoresistive sensor can determine and monitor the position of the pressure point on a discrete surface. Thus far, the adopted example illustrates the suitability of 3D printed piezoresistive sensors for strain-sensing applications in healthcare (e.g., gesture recognition or body movements). In more detail, activating the electrical signal along the lower sensitive direction can provide a warning about sprains. In other fields, such as robotics, such a sensor could be used for haptic perception to distinguish objects with different consistencies in the food industry (soft and hard food). In the case of soft food, a large movement would be detected along the 0° and 90° directions, while in the case of hard food, small movements of the robot would be detected by only a 90° direction signal. In a field such as transport (automotive or aeronautics), 3D printed sensors could be applied as real-time feedback structural health monitoring sensors to identify small damages, which in the long run can propagate and lead to much more serious failure for the material. In this last case, the detection could be used as an alarm to prevent irreversible failure.

## 4. Conclusions

In conclusion, this work demonstrates the potential of CNT-filled ABS sensors for applications requiring precise deformation monitoring, highlighting the influence of 3D printing parameters on sensor performance. The alignment of CNTs along the printing direction further boosts conductivity. Tensile and fatigue tests reveal that the sensor’s mechanical response varies with CNT concentration and raster angle. Samples printed at 0° demonstrate higher conductivity and mechanical strength than those printed at 90°. The sensor exhibits high sensitivity to strain, with gauge factor (G.F.) values ranging from 3.65 to 29.8 depending on the printing direction and CNT concentration. The 90° printed samples show greater sensitivity, especially at lower CNT concentrations. Creep tests indicate that the material’s permanent strain increases with higher loads, with the 90° printed samples showing higher residual resistance, evidencing points of weakness.

This paper also demonstrates the development and validation of a novel two-dimensional strain sensor capable of simultaneously measuring deformations on a plane with high sensitivity and a broad range of allowable deformations. The innovative cross-shaped design, produced using 3D printing techniques with a consistent raster angle, ensures precise detection of deformations in both the X- and Y-directions. The dual-element configuration allows for differentiated sensitivity, facilitating accurate monitoring of minor and significant deformations. This sensor’s adaptability, ease of calibration, and cost-effective production make it a valuable tool for various practical applications, including structural health monitoring and pressure point detection in composite structures. In this case, adhesion and delamination problems, owing to the technical complexity of the manufacturing process, could arise that compromise the functionality and reliability of the strain sensor. This aspect could be the objective of future research activity. A further step forward would be the development of three-dimensional strain sensors. The sensor proposed herein, able to detect strain in different directions in the plane, paves the way for the future perspective development of multiplane strain sensors by exploiting the advantages of the free-form 3D printing process.

## Figures and Tables

**Table 1:** Comparison among the most commonly used thermoplastic materials in the FFF process on their printability and mechanical properties.

	PLA			ABS			PA			PC			PET			TPU		
	L	M	H	L	M	H	L	M	H	L	M	H	L	M	H	L	M	H
Ease of printing			x		x			x			x			x		x		
Visual quality		x			x			x			x			x			x	
Max Stress		x			x			x				x		x			x	
Elongation at break	x			x				x		x			x					x
Impact resistance	x				x			x			x			x				x
Layer adhesion		x			x		x				x			x			x	
Heat resistance	x					x	x					x		x			x	

## Data Availability

The data presented in this study are available upon request from the corresponding author due to privacy reasons.

## Supplementary Materials

The following supporting information can be downloaded at: https://www.mdpi.com/article/10.3390/nano14211761/s1, Table S1: 3D Printing parameters; Table S2: Parameters set for acquiring TUNA current images; Figure S1. TUNA images at similar magnitude of: (a) the spooled filament and (b) the single printed filament; Figure S2. Scheme of the effect of printing direction and filler concentration on the tunneling distance dominating strain sensitivity.
