# Supplementary material for: Three-Dimensional Printed Nanocomposites with Tunable Piezoresistive Response
Source: Nanomaterials (Basel). 2024 Nov 2;14(21):1761. doi: 10.3390/nano14211761 (PMC11547783; doi:10.3390/nano14211761)
Supplement: Supplementary file 1 [file nanomaterials-14-01761-s001.zip › nanomaterials-3234579-supplementary.pdf]

Article

Three-Dimensional Printed Nanocomposites with Tunable Piezoresistive Response

Francesca Aliberti [1,*], Liberata Guadagno [1], Raffaele Longo [1], Marialuigia Raimondo [1], Roberto Pantani [1], Andrea Sorrentino [2,*], Michelina Catauro [3] and Luigi Vertuccio [3,*]

[1] Department of Industrial Engineering, University of Salerno, Via Giovanni Paolo II, 84084 Fisciano, Italy; lguadagno@unisa.it (L.G.); rlongo@unisa.it (R.L.); mraimondo@unisa.it (M.R.); rpantani@unisa.it (R.P.)
[2] Institute for Polymers, Composites, and Biomaterials (IPCB-CNR), Via Previati n. 1/E, 23900 Lecco, Italy
[3] Department of Engineering, University of Campania "Luigi Vanvitelli", Via Roma 29, 81031 Aversa, Italy; michelina.catauro@unicampania.it
* Correspondence: faliberti@unisa.it (F.A.); andrea.sorrentino@cnr.it (A.S.); luigi.vertuccio@unicampania.it (L.V.)

Keywords: 3D printed nanocomposites; piezoresistive response; printing direction; tunable gauge factor; two-dimensional sensor

Sample preparation

Table S1 reports the printing parameters, common to all printed samples, set in the Slic3r Prusa Edition (Prusa Research) software.

Table S1: 3D Printing parameters

Parameters	Specifics
Nozzle diameter	0.4 mm
Deposition width	0.4 mm
Layer thickness	0.2 mm
Infill density	100%
Platform temperature	80 °C
Extrusion temperature	250 °C
Printing speed	80 mm/s

Morphological characterization: SEM and TUNA analyses

SEM micrographs of the samples were acquired using SEM LEO 1525 (Carl Zeiss SMT AG, Oberkochen, Germany). Before the SEM investigation, the printed samples were subjected to a procedure to remove part of the polymeric matrix using an oxidizing solution (etching solution). The etching reagent was prepared by stirring 1.0 g potassium permanganate in a mixture of 95 ml sulfuric acid (95–97 %) and 48 ml orthophosphoric acid (85 %). The samples were immersed in the fresh etching reagent at room temperature and agitated for 24 hours. The samples were subsequently washed using a cold mixture of two parts by volume of concentrated sulfuric acid and seven parts water. The samples were washed with 30 % aqueous hydrogen peroxide to remove manganese dioxide. The samples were finally washed with distilled water and maintained under vacuum for 2 days. The as-treated printed samples were first fractured in liquid nitrogen to ensure no distortion during the rupture and then covered with a 25 0 - Å thick gold film using a sputter coater (Aga r mod. 10 8 A).

As regards AFM-TUNA investigation, the etched 3D-printed samples were directly investigated without the need for coating since the goal of this method is to track the conductive paths present in the samples due to the presence of conductive filler such as carbon nanotubes of the present article.

Table S2 summarizes the optimized parameters for acquiring TUNA current images.

Table S2: Parameters set for acquiring TUNA current images

Parameters	Specifics
DC bias	3 V
Current sensitivity	1 pA/V
Scan rate	0.500 Hz
Integral gain	2.000
Proportional gain	5.000
Sample lines per rump	512

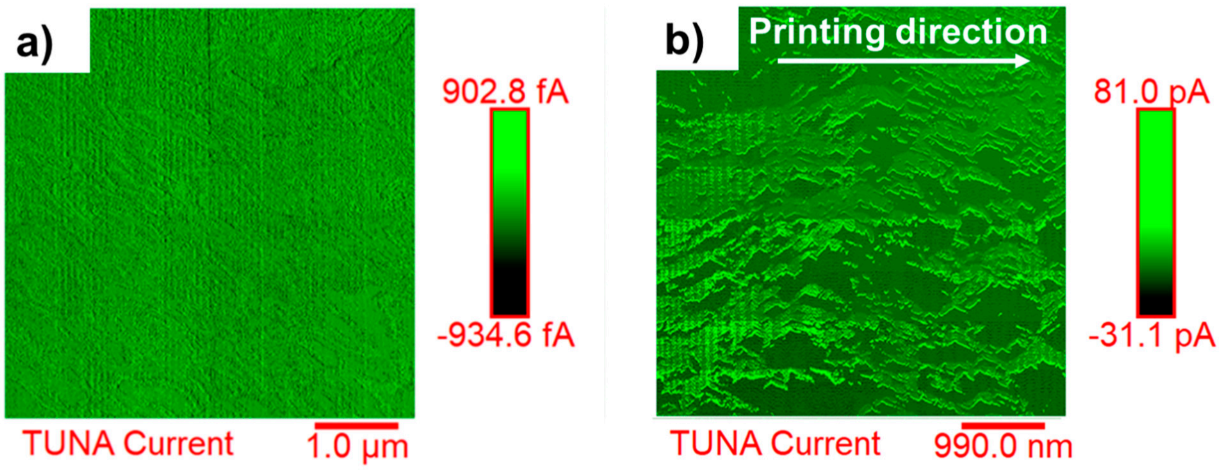

Figure S1. TUNA images at similar magnitude of: a) the spooled filament and b) the single printed filament.

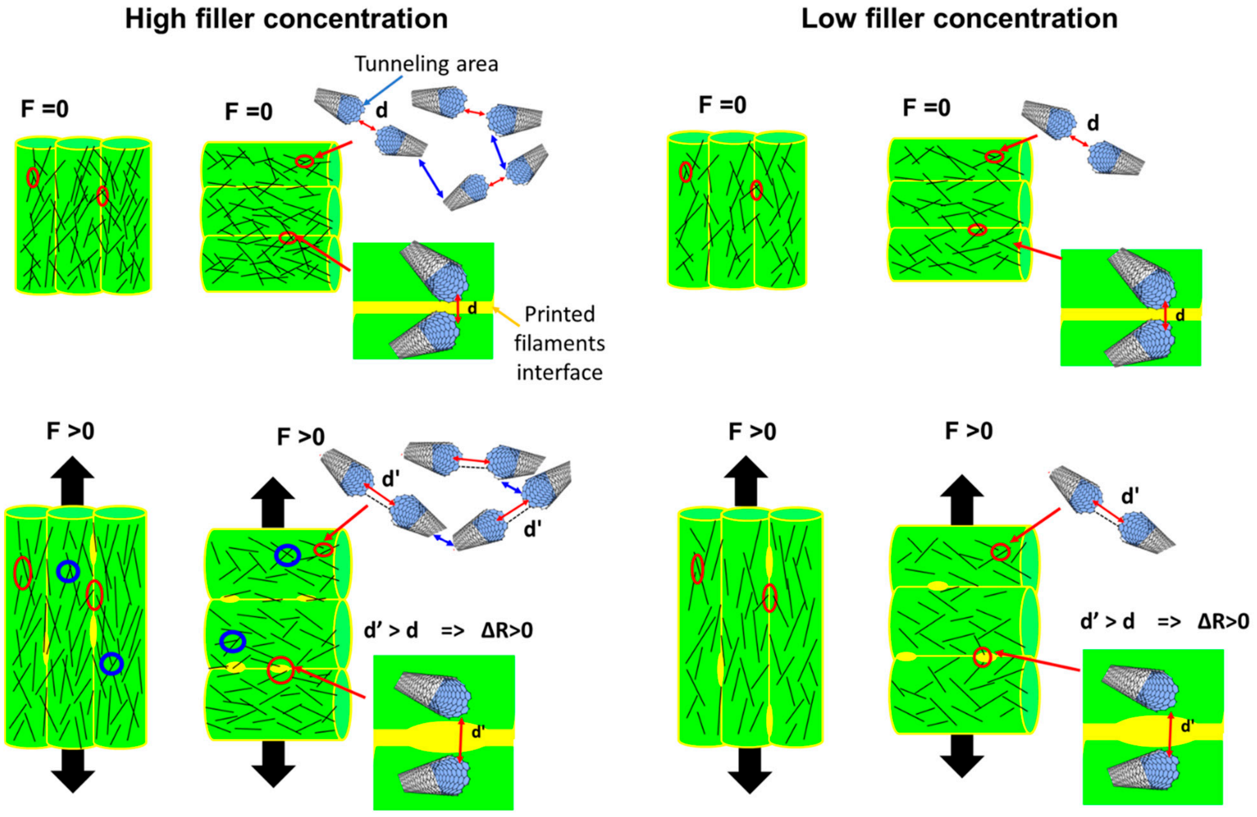

Figure S2. Scheme of the effect of printing direction and filler concentration on the tunneling distance dominating strain sensitivity.
